# Lateral Gene Transfer Acts As an Evolutionary Shortcut to Efficient C_4_ Biochemistry

**DOI:** 10.1093/molbev/msaa143

**Published:** 2020-06-10

**Authors:** Chatchawal Phansopa, Luke T Dunning, James D Reid, Pascal-Antoine Christin

**Affiliations:** 1 Department of Animal and Plant Sciences, University of Sheffield, Sheffield, United Kingdom; 2 Department of Chemistry, University of Sheffield, Sheffield, United Kingdom

**Keywords:** adaptation, C_4_ photosynthesis, horizontal gene transfer, positive selection, Poaceae

## Abstract

The adaptation of proteins for novel functions often requires changes in their kinetics via amino acid replacement. This process can require multiple mutations, and therefore extended periods of selection. The transfer of genes among distinct species might speed up the process, by providing proteins already adapted for the novel function. However, this hypothesis remains untested in multicellular eukaryotes. The grass *Alloteropsis* is an ideal system to test this hypothesis due to its diversity of genes encoding phosphoenolpyruvate carboxylase, an enzyme that catalyzes one of the key reactions in the C_4_ pathway. Different accessions of *Alloteropsis* either use native isoforms relatively recently co-opted from other functions or isoforms that were laterally acquired from distantly related species that evolved the C_4_ trait much earlier. By comparing the enzyme kinetics, we show that native isoforms with few amino acid replacements have substrate *K*_M_ values similar to the non-C_4_ ancestral form, but exhibit marked increases in catalytic efficiency. The co-option of native isoforms was therefore followed by rapid catalytic improvements, which appear to rely on standing genetic variation observed within one species. Native C_4_ isoforms with more amino acid replacements exhibit additional changes in affinities, suggesting that the initial catalytic improvements are followed by gradual modifications. Finally, laterally acquired genes show both strong increases in catalytic efficiency and important changes in substrate handling. We conclude that the transfer of genes among distant species sharing the same physiological novelty creates an evolutionary shortcut toward more efficient enzymes, effectively accelerating evolution.

## Introduction

The evolution of novel traits usually involves the co-option of preexisting genes, which were previously used for different functions (True and Carroll 2002; Jiggins et al. 2017; Fernández and Gabaldón 2020). These genes are often subsequently modified in terms of their expression pattern and/or properties of the encoded enzymes, the extent of which depends on the strength of selection (Toprak et al. 2012; Karageorgi et al. 2019). Mutations required to trigger certain new functions are often restricted to a subset of codon positions, and epistasis can restrict the order in which they can occur (Weinreich et al. 2006; Blount et al. 2012; Studer, Christin, et al. 2014; Kumar et al. 2017; Yang et al. 2019).  Because of these complexities, the modification of genes for a new function can require protracted periods of selection, the length of which depends on the mutation rate and demography of the species (Desai et al. 2007; Neher et al. 2010). The transfer of genes among species, via hybridization or lateral gene transfer (LGT), can bypass these extended periods of gradual evolution and boost evolutionary innovation (Ochman et al. 2000; Jain et al. 2003; Arnold and Kunte 2017; Hall et al. 2017). However, the impact of interspecific gene transfer on the speed of adaptation is difficult to directly compare with the iterative adaptation of co-opted native genes in complex multicellular organisms.

C_4_ photosynthesis offers a tractable system to study the evolutionary paths to new functions. This complex trait, which combines anatomical and biochemical modifications to increase productivity in tropical conditions (Hatch 1987; Atkinson et al. 2016), has evolved >60 times independently in flowering plants (Sage et al. 2011, 2012). All known C_4_ genes were present in the non-C_4_ ancestors, and their co-option involved a massive increase in their expression in specific leaf compartments, followed in some cases by kinetic adaptation of the encoded enzymes (Tausta et al. 2002; Engelmann et al. 2003; Gowik et al. 2004; Tanz et al. 2009; Aubry et al. 2011; Moreno-Villena et al. 2018; Alvarez et al. 2019; DiMario and Cousins 2019). In particular, the key C_4_ enzyme phosphoenolpyruvate carboxylase (PEPC) is highly expressed in all C_4_ plants, and the C_4_ forms of this enzyme differ from their non-C_4_ homologs in their affinities for the substrates as well as their sensitivity to inhibitors (Ting and Osmond 1973; Bauwe and Chollet 1986; Svensson et al. 1997; Gowik et al. 2006; Paulus et al. 2013; DiMario and Cousins 2019). Phylogeny-based sequence comparisons have shown that C_4_-specific genes for PEPC underwent numerous adaptive amino changes that were repeated among distant lineages (Christin et al. 2007; Besnard et al. 2009; Paulus et al. 2013; Rosnow et al. 2015). Although the kinetic effects of these mutations remain generally unknown (for exceptions, see Bläsing et al. 2000; Paulus et al. 2013; DiMario and Cousins 2019), the convergence of these C_4_-related mutations suggests that the adaptation of PEPC for the C_4_ context is similarly constrained in divergent C_4_ lineages. Importantly, although most C_4_-specific PEPCs originated via novel mutations that followed the co-option of native non-C_4_ genes, several instances of interspecific transfers of C_4_ PEPC have been reported (Besnard et al. 2009; Christin, Edwards, et al. 2012; Christin, Wallace, et al. 2012).

**Fig. 1.**
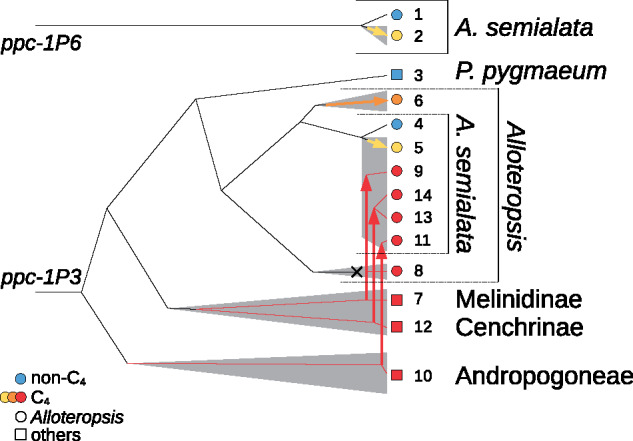
History of genes encoding PEPC in *Alloteropsis*. This schematic shows previously inferred relationships among the genes for PEPC analyzed (Christin, Edwards, et al. 2012; Dunning et al. 2017). Branching depths are proportional to estimated divergence times. C_4_ lineages are represented by gray areas, and arrows pointing to the tips represent modifications for the C_4_ function, whereas vertical arrows indicate interspecific gene transfers. The native copy of accession 8 (*A. cimicina*) was pseudogeneized, an event indicated with a cross. Genes are numbered as in table 1.

In grasses, the genus *Alloteropsis* includes plants that use C_4_ photosynthesis and others that lack the trait, sometimes within the same species (Ibrahim et al. 2009; Dunning et al. 2017). The C_4_ accessions of *Alloteropsis* use various PEPC genes for their C_4_ pathway, some of which were co-opted from other functions, whereas others were laterally acquired from distant C_4_ lineages (fig. 1; Christin, Edwards, et al. 2012; Dunning et al. 2017). Two different native non-C_4_ PEPC genes were co-opted by geographically isolated populations of *Alloteropsis semialata* (fig. 1 and table 1), which have undergone relatively few modifications since the trait evolved as evidenced by their high similarity to PEPC orthologs from non-C_4_  *A. semialata*, and a lack of the convergent amino acid replacements observed in older C_4_ lineages (Christin, Edwards, et al. 2012; Dunning et al. 2017). The sister species *A. angusta*, which likely evolved the C_4_ trait earlier, uses a native gene for PEPC co-opted from other functions that has undergone more amino acid replacements (fig. 1; Christin, Edwards, et al. 2012; Dunning et al. 2017). By contrast, several populations of *A. semialata* and *A. cimicina* use one of three PEPC genes that were laterally acquired from distantly related C_4_ lineages and likely replaced the co-opted native copies (fig. 1; Christin, Edwards, et al. 2012; Dunning et al. 2017). Because these other genes had spent millions of years within C_4_ plants before the transfer (fig. 1), they had been adapted for the C_4_ context (Christin et al. 2007; Christin, Edwards, et al. 2012). The unrivalled diversity of PEPC isoforms in *Alloteropsis* offers a unique opportunity to assess the biochemical changes conferred by interspecific transfers as opposed to adapting co-opted native genes.

**Table 1.** Isoform Sampling and Gene Information.

Index	Species	Accession^a^	Gene Lineage^b^	Category	Source
1	*Alloteropsis semialata*	RSA5-03	*ppc-1P6*	Non-C_4_	Synthesized
2	*Alloteropsis semialata*	TPE1-10	*ppc-1P6*	Native co-opted 1	Isolated^c^
3	*Panicum pygmaeum*	—	*ppc-1P3*	Non-C_4_	Moody et al. (forthcoming)
4	*Alloteropsis semialata*	RSA5-03	*ppc-1P3*	Non-C_4_	Synthesized
5	*Alloteropsis semialata*	MAD1-03	*ppc-1P3*	Native co-opted 2	Isolated^d^
6	*Alloteropsis angusta*	AANG4-8	*ppc-1P3*	Native co-opted 2	Synthesized
7	*Megahyrsus maximus*	—	*ppc-1P3_M*	Donor_M	Synthesized
8	*Alloteropsis cimicina*	—	*ppc-1P3_LGT:M*	LGT:M	Isolated^e^
9	*Alloteropsis semialata*	TAN4-08	*ppc-1P3_LGT:M*	LGT:M	Synthesized
10	*Themeda triandra*	—	*ppc-1P3_A*	Donor_A	Synthesized
11	*Alloteropsis semialata*	AUS1-01	*ppc-1P3_LGT:A*	LGT:A	Synthesized
12	*Setaria barbata*	—	*ppc-1P3_C*	Donor_C	Synthesized
13	*Alloteropsis semialata*	RSA3-01	*ppc-1P3_C*	LGT:C	Isolated^f^
14	*Alloteropsis semialata*	RSA4-01	*ppc-1P3_LGT:C*	LGT:C	Isolated^f^

a Accession names as in Dunning, Olofsson, et al. (2019).

b Genes named as in Bianconi et al. (2018) (M, Melinidinae; C, Cenchrinae; A, Andropogoneae).

c 5′ primer = AATAGCTAGCATGGCGGGGAAG (*Nhe*I), 3′ primer = AATACTCGAGTTAACCAGTGTT (*Xho*I).

d 5′ primer = AATACATATGGCGGCGTCC (*Nde*I), 3′ primer = AATAAAGCTTCTAGCCCGTGTT (*Hind*III).

e 5′ primer = AATACATATGGCGACCTCG (*Nde*I), 3′ primer = AATAGCGGCCGCCTAGCCCGTGTT (*Not*I).

f 5′ primer = AATACATATGGCGGAGAAG (*Nde*I), 3′ primer = AATAGCTAGCTAGCCAGTGTT (*Nhe*I).

In this work, we test the hypothesis that interspecific gene transfer provides an evolutionary shortcut to gene adaptations that would otherwise be achieved after a long period of selection on novel mutations. First, we establish the evolutionary trajectory of co-opted native PEPC enzymes within *Alloteropsis* by comparing the PEPC proteins of non-C_4_ and C_4_ accessions without any LGT PEPC. Second, we characterize genes from older C_4_ lineages that have numerous amino acid changes to test the hypothesis that they encode enzymes with drastically altered biochemical phenotypes when compared with non-C_4_ ancestors. Finally, we compare the properties of the enzymes encoded by the laterally acquired genes of *Alloteropsis* with the native copies of both *Alloteropsis* and the donor groups, to determine whether the transfers provided an evolutionary shortcut, and whether any further modifications of the kinetic properties happened after the transfers. Coupled with phylogenetic analyses of coding sequences, this work provides new insights into the evolutionary paths to new biochemical functions in plants, and the impact of gene transfers on physiological adaptations.

## Results

### Phylogenetic Analyses Confirm Different Amounts of Amino Acid Changes

**Fig. 2.**
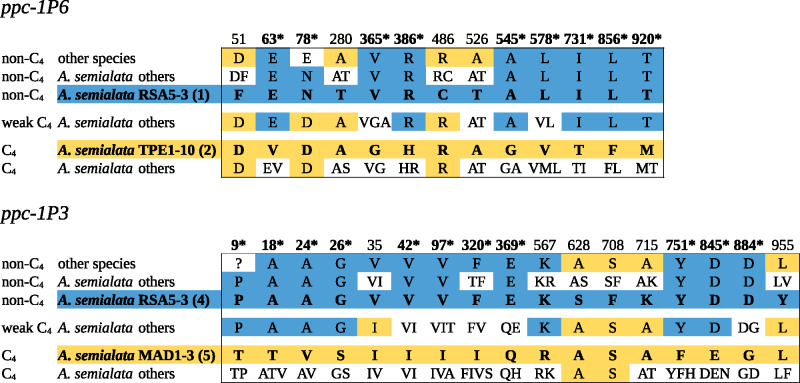
Amino acid variation of genes co-opted by C_4_  *Alloteropsis semialata*. For each of the two native gene lineages co-opted by *A. semialata* (*ppc1-P3* and *ppc-1P6*), the amino acid residues differing between the non-C_4_ and C_4_ cloned genes (names of accessions indicated with numbers in parentheses corresponding to those in fig. 1 and table 1) are shown, in blue for the non-C_4_ and yellow for the C_4_ forms. Homologous residues are reported in decreasing frequency for non-C_4_ orthologous of *A. semialata* and other species, genes of *A. semialata* with a weak C_4_ pathway (see Dunning et al. 2017), and other C_4_  *A. semialata*. When fixed within a group, the residues are colored as the cloned gene presenting the same residue. Positions are indicated on the top, numbered based on *Zea mays* sequence CAA33317. Asterisks highlight positions with novel mutations in the C_4_ group.

Genes from *Alloteropsis* were placed within the six distinct lineages of *ppc-1* as expected (supplementary fig. S1, Supplementary Material online; Dunning et al. 2017). The phylogeny inferred from *ppc-1P6* matched the species tree, with *A. angusta* genes sister to *A. semialata*, and the non-C_4_ individuals branching first within *A. semialata* (supplementary fig. S1, Supplementary Material online). Most amino acid replacements occurred on the two branches leading to groups of C_4_  *A. semialata*, one of which encompasses mainly Asian accessions, whereas the other one includes only African accessions. Many of these genes are pseudogenes, as evidenced by mutations disrupting the reading frame (supplementary fig. S1, Supplementary Material online). However, functional copies are detected in the individuals previously shown to use these genes for their C_4_ pathway (i.e., TPE1-10, BUR1-02, and RSA4-01; Dunning et al. 2017). The cloned variants of the C_4_ (from TPE1-10) and non-C_4_ (from RSA5-03) forms of *ppc-1P6* differ by 13 amino acids (table 2), and in four cases, the C_4_ form harbors the ancestral residue as observed in other non-C_4_ species (sites 51, 280, 486, and 526; fig. 2). Of the nine replacements that represent novel mutations in the C_4_, only one is fixed among C_4_ accessions (site 78; fig. 2).

**Table 2.** Pairwise Number of Amino Acid Differences between Analyzed Sequences^a^.

Category	Isoform	1	2	3	4	5	6	7	8	9	10	11	12	13	14
Non-C_4_	1. RSA5-03	0													
Native co-opted 1	2. TPE1-10	13	0												
Non-C_4_	3. *Panicum pygmaeum*	154	153	0											
Non-C_4_	4. RSA5-03	161	161	20	0										
Native co-opted 2	5. MAD1-03	164	164	29	17	0									
Native co-opted 2	6. *Alloteropsis angusta*	187	185	69	59	64	0								
Donor_M	7. *Megahyrsus maximus*	224	223	148	149	147	152	0							
LGT:M	8. *Alloteropsis cimicina*	218	217	142	143	137	147	39	0						
LGT:M	9. TAN4-08	221	220	146	147	140	147	47	22	0					
Donor_A	10. *Themeda triandra*	234	234	180	185	184	178	190	188	190	0				
LGT:A	11. AUS1-01	236	235	181	186	185	179	191	189	191	3	0			
Donor_C	12. *Setaria barbata*	218	217	147	148	151	148	153	159	162	190	193	0		
LGT:C	13. RSA3-01	220	219	149	150	153	150	155	161	164	192	195	2	0	
LGT:C	14. RSA4-01	220	219	149	150	153	150	155	161	164	192	195	2	0	0

a Sequences are numbered as in table 1; differences between sequences belonging to the same group (see supplementary fig. S1, Supplementary Material online) are shaded in gray.

The phylogeny based on the native copy of *ppc-1P3* also recovered the expected relationships among species and accessions. An abundance of amino acid replacements occurred on the branch leading to the *A. cimicina* gene, which is a pseudogene (supplementary fig. S1, Supplementary Material online), and to a lesser extent on the branch leading to *A. angusta* genes, which are functional and used by this species for the C_4_ pathway (Dunning et al. 2017). Within *A. semialata*, many *ppc-1P3* genes from C_4_ accessions are pseudogenes, and few amino acid mutations are observed (table 2), mainly on branches leading to genes used by some C_4_ accessions (e.g., MAD1-03, TPE1-10, and BUR1-02; Dunning et al. 2017). The cloned variants of the C_4_ (from MAD1-03) and non-C_4_ (from RSA5-03) genes differ by a total of three amino acid deletions and 17 amino acid substitutions, four of which represent novel mutations in the non-C_4_ form (sites 628, 708, 715, and 955) and an extra two sites are variable among non-C_4_ accessions (sites 35 and 567, fig. 2). All of the 11 sites representing new mutations in the C_4_ forms are polymorphic among C_4_ accessions (fig. 2), and in many cases within individuals. Three of these 11 amino acid substitutions are also observed in *A. angusta* (sites 18, 320, and 369), but many more substitutions occurred in this species (table 2). Indeed, the cloned C_4_ gene from *A. angusta* differs from the cloned non-C_4_ variant from *A. semialata* by 59 amino acid substitutions, one insertion and one deletion (supplementary data set 1, Supplementary Material online). Nine of the amino acid residues specific to the C_4_ form of *A. angusta* are among the 21 previously reported as convergent among C_4_ lineages of grasses (positions 531, 577, 579, 780, 794, 572, 813, 502, 665; Christin et al. 2007; Christin, Edwards, et al. 2012).

The close relationships between genes laterally acquired by *Alloteropsis* and some other groups of grasses are confirmed (supplementary fig. S1, Supplementary Material online). The *ppc-1P3_LGT:C* gene of *A. semialata* is almost identical to that of *Setaria barbata* (two differences between the cloned genes; table 2), with very few amino acid differences among *A. semialata* accessions (supplementary fig. S1, Supplementary Material online). A great similarity is also observed between the *ppc-1P3_LGT:A* gene of *A. semialata* and *Themeda triandra* (three amino acid differences between the cloned genes; table 2 and supplementary fig. S1, Supplementary Material online). By contrast, the *ppc-1P3_LGT:M* genes of *Alloteropsis* are relatively diverged from all sequences available for the group of donors, and highly divergent copies are observed within *A. cimicina* (table 2 and supplementary fig. S1, Supplementary Material online). Frequent amino acid replacement in the *ppc-1P3_LGT:M* genes also occurred within *A. semialata*, and although several copies are pseudogenes, functional versions are observed in accessions previously shown to use this gene for their C_4_ pathway (e.g., BUR1-02 and TAN4-08; Dunning et al. 2017).

### Gradual Modifications Following the Co-option of Native Genes

**Fig. 3.**
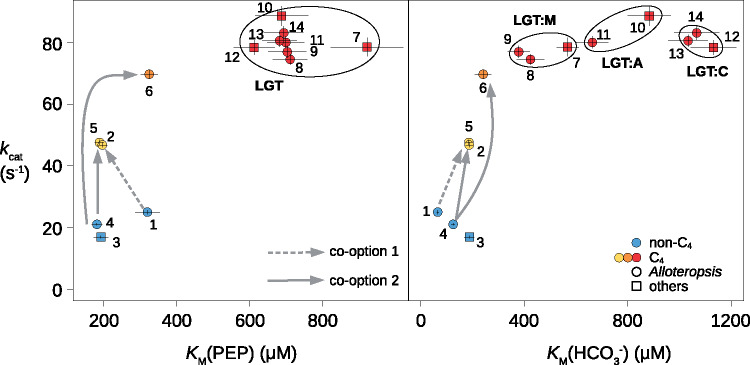
Comparison of kinetic parameters. Measured values are shown for the 14 assayed enzymes, with error bars showing SDs. Changes following the co-option of native genes are indicated with gray arrows, and ellipses indicate genes involved in lateral gene transfers (LGT). Genes are numbered as in table 1.

**Fig. 4.**
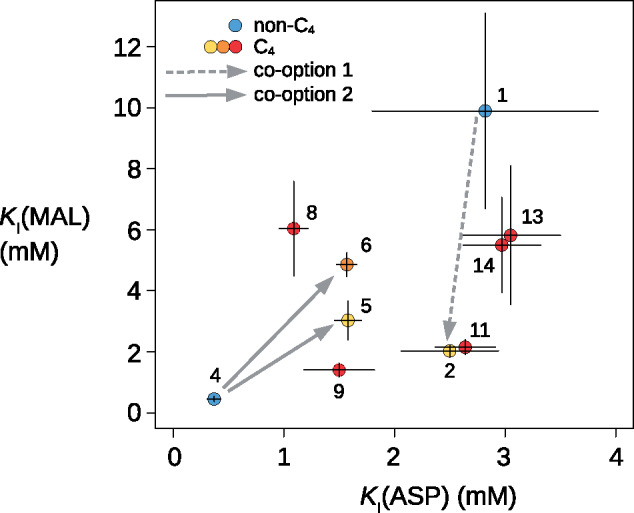
Comparison of sensitivity to inhibitors. Measured values are shown for different forms of PEPC from *Alloteropsis*, with error bars showing SDs. Genes are numbered as in table 1.

We cloned and synthesized proteins encoded by a total of 14 genes from *Alloteropsis* accessions and related grasses (table 1), which capture a diversity of origins of C_4_ PEPC (fig. 1). The enzyme encoded by the non-C_4_  *ppc-1P6* of *A. semialata* has a low *K*_M_ for both substrates (PEP and ${\text{HCO}}_{3}^{-}$) and a low *k*_cat_ (isoform 1 in fig. 3; supplementary table S1, Supplementary Material online). In comparison, the enzyme encoded by the co-opted native ortholog (isoform 2) has a decreased *K*_M_(PEP), an increased *K*_M_(${\text{HCO}}_{3}^{-}$), and an increased *k*_cat_ (1.87-fold; fig. 3 and supplementary table S1, Supplementary Material online). The co-option of native *ppc-1P6* was therefore followed by an increased catalytic efficiency and small alterations of the *K*_m_ for each substrate. The non-C_4_ enzyme encoded by *ppc-1P6* (isoform 1) showed the lowest sensitivities to both malate and aspartate (two molecules that are produced downstream in the C_4_ pathway) of all assayed enzymes, and the co-opted native enzyme (isoform 2) showed a markedly increased sensitivity to malate inhibition (fig. 4).

The enzymes encoded by the non-C_4_  *ppc-1P3* of *A. semialata* and the close relative *Panicum pygmaeum* are kinetically very similar (isoforms 3 and 4, respectively; supplementary table S1, Supplementary Material online). They present the lowest *k*_cat_ and *K*_M_(PEP) of all isoforms analyzed here, and rank among the lowest *K*_M_(${\text{HCO}}_{3}^{-}$) (fig. 3). In terms of kinetics, the enzyme encoded by non-C_4_  *ppc-1P3* (isoforms 3 and 4) are similar to that encoded by non-C_4_  *ppc-1P6* (isoform 1), despite >100 My of divergence and many amino acid differences (table 2). However, enzymes encoded by non-C_4_  *ppc-1P3* (isoforms 3 and 4) and *ppc-1P6* (isoform 1 ) differ strongly in terms of their sensitivity to inhibitors, which exhibit the lowest and highest values, respectively (fig. 4). The enzyme encoded by the native *ppc-1P3* co-opted for C_4_ photosynthesis by *A. semialata* (isoform 5) is very similar to those encoded by the non-C_4_ orthologs (isoforms 3 and 4) in terms of *K*_M_ for both substrates, but has a markedly elevated *k*_cat_ (2.26-fold higher; fig. 3) and reduced sensitivities to both malate and aspartate (fig. 4). The *k*_cat_ and sensitivity to inhibition change in the same direction, but are more marked in the co-opted native form from *A. angusta* (isoform 6; figs. 3 and 4). However, the *K*_M_(PEP) is ∼1.7× larger in the co-opted native form from *A. angusta* (isoform 6) as compared with enzymes encoded by both C_4_ and non-C_4_ orthologs from *A. semialata* (isoforms 4 and 5, fig. 3). These results suggest that the co-option of native *ppc-1P3* was followed by rapid changes in *k*_cat_ and sensitivity to inhibition, and later by modifications of the *K*_m_(PEP).

Overall, enzymes encoded by the non-C_4_ paralogs *ppc-1P3* (isoforms 3 and 4) and *ppc-1P6* (isoform 1) differ in their kinetic properties, as expected from their long divergence (fig. 1 and table 2). The changes consequently happened in slightly different directions after the co-option of the native *ppc-1P6* than following each co-option of native *ppc-1P3* (figs. 3 and 4). However, the kinetic parameters of the enzymes encoded by co-opted native *ppc-1P3* (isoform 5) and *ppc-1P6* (isoform 2) from *A. semialata* are almost identical (figs. 3 and 4), indicating rapid convergence.

### Laterally Acquired Genes Are Highly Divergent from the Non-C_4_ Forms

The three laterally acquired versions (isoforms 8 + 9, 11, and 13 + 14) are massively different from the native C_4_ and non-C_4_ enzymes (isoforms 1–6), but are similar to those of the close relatives of the donors (isoforms 7, 10, and 12). All laterally acquired versions (isoforms 8, 9, 11, 13, and 14) have strikingly convergent *k*_cat_ and *K*_M_(PEP), the latter of which are 1.8/3.9-fold higher than those of the native versions (isoforms 1–6; fig. 3 and supplementary table S1, Supplementary Material online). Their *K*_M_(${\text{HCO}}_{3}^{-}$) are more variable, but in all cases above those of the co-opted native isoforms (isoforms 2, 5, and 6), and each *A. semialata* copy clusters with its donor (fig. 3). The sensitivity to inhibitors of the laterally acquired isoforms overlaps with those of the co-opted native C_4_ isoforms (fig. 4). Because the LGT replaced the co-opted native versions of *A. semialata* (isoforms 2 and 5; Olofsson et al. 2016; Dunning et al. 2017), the LGTs have led to a >1.5-fold increase of *k*_cat_, a >3.1-fold increase of *K*_M_(PEP), and a >2-fold increase of *K*_M_(${\text{HCO}}_{3}^{-}$), without consistent modifications of the sensitivity to inhibitors (figs. 3 and 4).

## Discussion

### Rapid Increase in Catalytic Efficiency after the Co-option of Native PEPC for C_4_ Photosynthesis

Most C_4_ lineages emerged between 5 and 30 Ma, so that the early events of the photosynthetic transitions are blurred by the accumulation of unrelated mutations (Heyduk et al. 2019). As a comparatively young C_4_ lineage (<3 Ma; Lundgren et al. 2015), C_4_ accessions of *A. semialata* represent an excellent system to pinpoint the exact modifications involved in the early emergence of a C_4_ physiology, as previously applied to anatomical traits and gene expression (Dunning, Moreno-Villena, et al. 2019; Lundgren et al. 2019). In the case of PEPC, the non-C_4_ enzymes encoded by *ppc-1P3* and *ppc-1P6* likely resemble the ancestral forms, as suggested by the limited number of amino acid changes in non-C_4_ plants (table 2 and supplementary fig. S1, Supplementary Material online), and the catalytic similarity between enzymes encoded by the non-C_4_  *ppc-1P3* of *A. semialata* and the more distantly related *P. pygmaeum* (figs. 3 and 4). The enzymes encoded by the non-C_4_ paralogs vary in their kinetic properties (figs. 3 and 4), as expected given their long divergence time (near the origin of monocots 140–160 Ma; Deng et al. 2016; Li et al. 2019). However, both isoforms present low *K*_M_ for the two substrates, as reported for other non-C_4_ isoforms (Dong et al. 1998; Bläsing et al. 2002; Gowik et al. 2006). This might confer rapid responses to small increases of substrate and therefore a tight regulation of the non-C_4_ function (O’Leary et al. 2011). Our comparative analyses show that the co-option of both native *ppc-1P3* and *ppc-1P6* was followed by swift changes to the catalytic efficiency and sensitivity to inhibitors, as observed in the C_4_  *A. semialata* (figs. 3 and 4). Reduced inhibition by the products of PEPC is likely required to allow the enzyme to function in the high-flux C_4_ pathway (Svensson et al. 1997, 2003), which leads to massively elevated concentrations of metabolites (Arrivault et al. 2017). Increased catalytic efficiency would directly impact the rate of the cycle providing a selective advantage to emerging C_4_ plants (Heckmann et al. 2013).

Although the causal mutations are not known, the characterized C_4_-specific native *ppc-1P3* and *ppc-1P6* of *A. semialata* differ from their respective non-C_4_ orthologs by few amino acids (table 2), some of which are also observed among non-C_4_ individuals, whereas almost all others are polymorphic within the C_4_ group (fig. 2). This suggests that the C_4_-specific properties might have emerged from standing genetic variation, after recombination generated amino acid combinations that altered the properties of the encoded enzyme in synergy. Many of the amino acid differences are moreover polymorphic within C_4_ individuals (fig. 2), which suggests that this process is ongoing, potentially as part of the functional diversification of the multiple copies that exist within some of these plants (Bianconi et al. 2018).

### Adaptation of the Protein Sequence Leads to Further Biochemical Changes

*Alloteropsis angusta* diverged from *A. semialata* ∼7 Ma (Lundgren et al. 2015; Dunning et al. 2017). Its native *ppc-1P3* shows signs of positive selection (Dunning et al. 2017), and it presents some of the amino acids that convergently evolved in older C_4_ lineages (Christin et al. 2007; Christin, Edwards, et al. 2012; Christin, Wallace, et al. 2012). This co-opted native gene can thus be considered as partially modified for the C_4_ context. Because some of the amino acid differences between the native C_4_ and non-C_4_ isoforms of *A. semialata* are also observed in *A. angusta*, it is possible that the adaptation of *A. angusta* PEPC for the C_4_ context initially followed the same path observed within *A. semialata*. In terms of enzyme phenotype, the C_4_ form from *A. angusta* is even less sensitive to malate than its native C_4_ ortholog from *A. semialata* (fig. 4). It moreover shows a higher catalytic efficiency (fig. 3), which suggests that initial large-effect changes as observed within *A. semialata* are then followed by further modifications in the same direction. In addition, the C_4_ isoform from *A. angusta* differs from both C_4_ and non-C_4_ native forms from *A. semialata* in its increased *K*_M_ for PEP (fig. 3). This change has been observed in other C_4_ lineages, but its physiological significance remains unknown (Ting and Osmond 1973; Bläsing et al. 2000; Gowik et al. 2006). One hypothesis is that it represents a side effect of selection for another property, such as reduced inhibition by malate or different affinity for ${\text{HCO}}_{3}^{-}$ (Svensson et al. 1997, 2003). Our study argues against this hypothesis as there is a lack of a correlation between these parameters and the *K*_M_ for PEP. Instead, it is likely that the increased *K*_M_ for PEP evolved in C_4_ plants to allow a tighter regulation when substrate concentrations are high (Ting and Osmond 1973; Svensson et al. 2003). Although this hypothesis remains to be tested, our data show that the amino acid replacements observed in the native *ppc-1P3* of *A. angusta* lead to a strengthening of the rapid changes observed in *A. semialata*, with further alterations of *K*_M_ for the substrates.

### Lateral Gene Transfer Provides a Shortcut to Adaptation

The enzymes encoded by genes laterally acquired from three different grass lineages representing two C_4_ origins (fig. 1) are highly similar in terms of their catalytic efficiency and affinity for PEP, which reflects convergence among the donor species (fig. 3). It is however clear from other studies that not all C_4_ PEPC have the exact same properties (Ting and Osmond 1973; Moody et al. forthcoming), and we suggest that the clustering of properties reflects a bias in the genes that successfully transferred into *Alloteropsis*.

Compared with the co-opted native isoform from *A. angusta*, the catalytic efficiency of the laterally acquired versions is only slightly higher (fig. 3). However, their *K*_M_ values are massively increased (fig. 3). We conclude that the trend observed in *A. angusta* was continued in other lineages, leading to enzymes with very high *K*_M_ for PEP in older C_4_ groups. The *K*_M_ for ${\text{HCO}}_{3}^{-}$ is also strongly increased in the laterally acquired isoforms, which is opposite to differences observed in other C_4_ systems (Bauwe 1986; DiMario and Cousins 2019; Moody et al. forthcoming). This might indicate that the optimal interaction with ${\text{HCO}}_{3}^{-}$ is context dependent. Indeed, the enzyme catalyzing ${\text{HCO}}_{3}^{-}$ production is essential in only some C_4_ plants (Studer, Gandin, et al. 2014), suggesting that the substrate is naturally abundant in others. In all cases, the laterally acquired genes show amplified differences with the non-C_4_ orthologs when compared with the co-opted native isoform of *A. angusta* (fig. 3). Because the co-opted native orthologs of *A. semialata* lack most C_4_-specific amino acid modifications, the laterally acquired genes generated an extreme jump in the enzyme catalytic properties (fig. 3). The integration of these isoforms in the C_4_ pathway of *A. semialata* therefore provided a direct shortcut, forgoing the long phase of adaptive evolution observed in *A. angusta* and other groups. We conclude that LGTs represent a highway to biochemical adaptation in plants.

The leaf anatomy and C_4_ biochemistry are similar between the donors of *ppc-1P3_LGT:A* and* ppc-1P3_LGT:C*, and *A. semialata* (Prendergast et al. 1987; Renvoize 1987; Dunning et al. 2017), which might explain why the transfers were not followed by significant modification to the encoded enzyme. The C_4_ phenotype is also similar between the donor of *ppc-1P3_LGT:M* and *A. cimicina*, which is the original recipient of the gene (Dunning et al. 2017). The *ppc-1P3_LGT:M* gene was subsequently introgressed from *A. cimicina* to *A. semialata* (Dunning et al. 2017), which despite being closely related markedly differ in their C_4_ anatomy (Dunning et al. 2017). Interestingly, this *A. semialata ppc-1P3_LGT:M* was replaced by *ppc-1P3_LGT:C* in several *A. semialata* accessions, and the former has been pseudogenized (supplementary fig. S1, Supplementary Material online; Olofsson et al. 2016). It is possible that the kinetic properties of the latter, including a larger *K*_M_ for ${\text{HCO}}_{3}^{-}$ and a reduced sensitivity to aspartate (figs. 3 and 4), were advantageous in *A. semialata*, a species whose C_4_ cycle relies on an aspartate shuttle (Dunning, Moreno-Villena, et al. 2019). We therefore suggest that the fit of the laterally acquired genes depends on the functional similarity between the donor and recipient species, making some evolutionary shortcuts more advantageous.

## Conclusions

The evolution of complex traits, such as C_4_ photosynthesis, involves the co-option of numerous genes, often requiring their subsequent modification to adapt the encoded enzymes for the new biochemical context. In the case of PEPC, the massive upregulation in expression of the non-C_4_ copies was followed by amino acid replacements that rapidly increased the catalytic efficiency and sensitivity to inhibitors of the enzyme. This process, evidenced within *A. semialata*, likely capitalized on standing genetic variation. The resultant enzyme was able to sustain a functioning C_4_ cycle, but was likely suboptimal and over time underwent secondary adaptations. This evolutionary process involved the fixation of novel mutations that are absent from non-C_4_ forms and therefore likely necessitated substantial evolutionary time, explaining why the co-opted native isoform from *A. angusta* presents only some of the characteristics of older C_4_ lineages. The interspecific transfer of genes already adapted to the C_4_ context in these older groups provided a shortcut to evolutionary adaptation, bringing in enzymes that directly improved the novel physiology. Our work therefore shows that LGTs among grasses generated a leap toward the adaptation of emerging physiologies. We predict that such successful transfers will be more prevalent in the case of genes requiring extensive adaptations, as is the case of PEPC for the C_4_ context.

## Materials and Methods

### Phylogenetic Analysis of the *ppc-1* Gene Family

We generated phylogenetic trees for different groups of the gene lineage *ppc-1* containing forms used for C_4_ photosynthesis by some *Alloteropsis* (Dunning et al. 2017). Sequences were obtained from published transcriptomes and genomes (Moreno-Villena et al. 2018; Dunning, Olofsson, et al. 2019) or retrieved from NCBI database. In addition, we also included data for *A. semialata* (AUS1-01 accession; Dunning, Olofsson, et al. 2019), *A. angusta* (AANG4-8; unpublished), *A. cimicina* (data from Dunning, Olofsson, et al. 2019 and assembled using the same method), and *T. triandra* (Dunning, Olofsson, et al. 2019). Apart from the chromosome-level assembly of *A. semialata*, these genomes were generated solely using short-read data and as a result, the assemblies are highly fragmented. We therefore had to assemble the *ppc-1* gene models from multiple contigs, and used *Setaria italica* and *Sorghum bicolor* sequences as a reference. We also generated gene models for two genes from a Zambian *A. semialata* accession (ZAM15-05-10) which were either truncated in AUS1-01 reference (*ppc-1P6*), or absent (*ppc-1P3_C*). Coding sequences were extracted from additional *Alloteropsis* short-read data sets as described in Dunning, Olofsson, et al. (2019). All gene models from each group of interest were then aligned using mafft v7.123b (Katoh and Standley 2013). For each group, a maximum likelihood phylogenetic tree was inferred using the third-codon positions to avoid biases due to convergent adaptive evolution. This was performed with PhyML v.21031022 (Guindon and Gascuel 2003) using the best substitution model identified using Smart Model Selection SMS v.1.8.1 (Lefort et al. 2017). Branch lengths were subsequently also estimated in amino acid substitution on the fixed topology using codeml v.4.7 (Yang 2007) with the M0 model.

### Isolation and Cloning of *ppc-1* Genes

Genes representing a diversity of origins (fig. 1 and table 1) were selected for detailed biochemical characterization. This included native copies co-opted for C_4_ photosynthesis, non-C_4_ forms of the native copies as well as C_4_ forms from species closely related to the putative donor for each laterally acquired gene (fig. 1 and table 1). To account for diversity within *Alloteropsis* two different variants were targeted for some genes (*ppc-1P3*, *ppc-1P3_LGT:M*, and *ppc-1P3_LGT:C*). Finally, a non-C_4_ ortholog from a close relative of *Alloteropsis* (*P. pygmaeum*) was included using a previously prepared plasmid (Moody et al. forthcoming).

Complete coding sequences corresponding to the most abundantly transcribed copies, as identified based on transcriptome analyses (Dunning et al. 2017; Dunning, Moreno-Villena, et al. 2019), were isolated by PCR from leaf cDNAs. RNA was extracted from mature leaves that had been exposed to 7 h of light, using the RNeasy Plant Mini Kit (Qiagen). The synthesis of cDNA was then performed using the MultiScribe Reverse Transcriptase (Applied Biosystems) and RT random primers, following the manufacturer’s instructions. Amplification was performed with the Q5 High-Fidelity DNA Polymerase (New England Biolabs), with primers corresponding to the 5′ and 3′ extremities of each targeted gene (table 1), as determined from previous transcriptomes (Dunning et al. 2017; Dunning, Moreno-Villena, et al. 2019). Each primer includes a digestion site before the start and after the stop codons (table 1), for follow-up cloning. The PCR mixture contained 1× Q5 Reaction Buffer, 200 μM dNTPs, 0.5 μM of each primer, ∼ 900 ng template cDNA, and 0.5 U Q5 DNA polymerase. A denaturing, annealing, and extension temperature of 98 °C (10 s), 57 °C (30 s), and 72 °C (3 min), respectively, were used in the PCR reactions over 35 cycles.

Successful PCR products were gel extracted using the QIAquick Gel Extraction Kit (Qiagen), and the purified products were digested with the appropriate restriction endonucleases (table 1). The digested products were ligated into pET-28a(+) expression vectors (Novagen), using a T4 DNA ligase (New England Biolabs). The vectors had been previously digested with the appropriate enzymes, so that genes were cloned in-frame with the T7 promoter, *lacO*, ribosome-binding site, and N-terminal hexa-Histidine tag. The cloned constructs were Sanger sequenced using the T7 promoter and terminator primers and compared with the transcriptome data to verify the identity of the cloned genes. For several genes, PCR amplification failed, potentially because of low gene expression. In other cases, the unavailability of live plants prevented RNA isolation. These genes were therefore synthesized by GeneArt (LifeTechnologies) and directly cloned into the pET100/D-TOPO expression vector for codon-optimized expression in *Escherichia coli*.

### Heterogeneous Expression and Purification of Recombinant PEPC

The 14 *ppc* constructs were used in the transformation of competent *E. coli* BL21λDE3 (Novagen) cells. Successfully transformed cells were selected for using either 50 mg ml^−1^ ampicillin (Sigma–Aldrich) or 30 mg ml^−1^ kanamycin (Sigma–Aldrich) depending on the plasmid vector. Bacterial cells were cultured in 2×TY media (1.6% [*w*/*v*] tryptone, 1% [*w*/*v*] yeast extract, 0.5% [*w*/*v*] NaCl, adjusted to pH 7.0 with NaOH and sterilized by autoclaving) at 25 °C with vigorous agitation and appropriate antibiotic added. At the mid-log phase (A_600_ = ∼0.6), the cultures were chilled at 4 °C for 1 h, then induced with 1 mM isopropyl β-d-1-thiogalactopyranoside (IPTG; filter-sterilized; Melford) at 16 °C for a further 39 h. Cells were harvested by centrifugation at 4 °C (10 min; 14,000 × *g*), resuspended in lysis buffer (0.2 M Tris–HCl, 0.5 M NaCl, pH 8.0, with either pefabloc SC or Roche complete mini [EDTA free] protease inhibitors at the manufacturers recommended concentrations), and disrupted using a French pressure cell press (Constant Systems). The suspension was clarified by two sequential centrifugations at 4 °C (31,000 × *g*) for 15 min and 30 min, and the supernatants were passed through a 0.45-μm filter (Millipore) before it was fractionated on a 1-ml His-Trap HP column (GE Healthcare) at 1 ml min^−1^ on the ÄKTA pure (GE Healthcare), which was preequilibrated in the Binding Buffer (0.2 M Tris–HCl, 0.5 M NaCl, 50 mM imidazole [Sigma–Aldrich], pH 8.0). After washing with 60× column volumes of Wash Buffer (0.2 M Tris–HCl, 0.5 M NaCl, 100 mM imidazole, pH 8.0), recombinant PEPC was gradient-eluted with Elution Buffer (0.2 M Tris–HCl, 0.5 M NaCl, 400 mM imidazole, pH 8.0). Fractions containing eluted protein were then pooled and desalted using a 5-ml HiTrap Desalting Column (GE Healthcare) that had been preequilibrated with Storage Buffer (0.2 M Tris–HCl, 50 mM NaCl, 10% [*v*/*v*] glycerol, pH 8.0). Upon elution, the purified protein, as judged pure by resolving on a 10% Mini-Protean TGX precast gel (Bio-Rad) via SDS–PAGE and Coomassie Blue (Sigma–Aldrich) staining, was snap-frozen in aliquots and stored at −80 °C. The concentration of PEPC was determined using a NanoDrop UV-Vis spectrophotometer (ThermoFisher) whereby the A_280_ measurements (subtracted by A_310_) were divided by the predicted extinction coefficient of the amino acid sequence of a PEPC fused to the N-terminal hexa-Histidine tag (according to the ProtParam tool on the ExPASy server; web.expasy.org/protparam/).

### Kinetic Analyses

Rates of PEPC catalyzed formation of oxaloacetate were measured spectroscopically by coupling to malate dehydrogenase where oxidation of the NADH cofactor can be monitored at 340 nm. Assays with a high, fixed, concentration of bicarbonate (${\text{HCO}}_{3}^{-}$) were observed using a FLUOstar plate reader (BMG Labtech) through a 340 ± 5 nm bandpass filter in absorbance mode with a reaction volume of 150 μl. Assays where bicarbonate concentrations were varied were observed at 340 nm using a Cary spectrophotometer (Agilent Technologies) in a 1-ml volume. All reactions were at 25 °C and followed for at least 15 min. NADH concentrations in the plate reader were determined using a standard curve. All assays were performed using three or more independently purified PEPC with three technical replicates. Initial rates were corrected for blank rates, determined in the absence of PEPC.

Assays typically contained 50 mM Tris–HCl (pH 7.4), 5 mM MgCl_2_, 6 Uml^−1^ malate dehydrogenase (porcine heart; Sigma), 0.2 mM NADH, 10 μM–5 mM PEP, 10 μM–10 mM KHCO_3_, and were initiated by addition of PEPC (2–9 nM, final concentration). When the concentration of bicarbonate was varied KCl was added to maintain a constant ionic strength, background bicarbonate was removed by extensive sparging with N_2_ and residual bicarbonate was determined by assay in the absence of added bicarbonate.

Inhibition parameters were determined for *Alloteropsis* genes at fixed bicarbonate (10 mM), variable PEP, and inhibitor (l-malate and l-aspartate) concentrations between 0 and 25 mM.

### Kinetic Data Analysis

Kinetic parameters were determined by nonlinear regression analysis in Igor Pro (Version 8; Wavemetrics Inc.). In the absence of inhibitor, data were analyzed with equation (1), where *K*_iA_*K*_B_ was held at 50 μM^2^ and with a correction factor for differences in activity between runs.

(1) $$v_{0}=(V_{\text{max}}\cdot \left[A\right]\cdot \left[B\right]/\;(\left[A\right]\cdot \left[B\right]+K_{A}\cdot \left[B\right]+K_{B}\cdot \left[A\right]+K_{\text{iA}}K_{B}).$$

Estimates of the SE values for *k*_cat_ (i.e., *V*_max_/[E]_T_) and the two *K*_m_ values (i.e., *K*_A_ and *K*_B_) were produced directly from the nonlinear regression analysis. 

Inhibition parameters (*K*_I_) were determined from secondary plots of (*k*_cat_/*K*_m_)^app^ against inhibitor concentration fitted to equation (2).

(2) $${\left(k_{\text{cat}}/K_{m}\right)}^{\text{app}}=\left(k_{\text{cat}}/K_{m}\right)/\left(1+\left[I\right]/K_{I}\right).$$

### In Vivo Enzymatic Assays

Enzymes purified from leaves of the plants used to isolate the genes were characterized to determine whether posttranscriptional modification affects the kinetic patterns. The plants were maintained under greenhouse conditions with supplementary lightings (Agrolux), temperature control (25 °C in the day and 20 °C at night; Mitsubishi Electric), and a light pollution screen (CambridgeHOK) at The Arthur Willis Environment Centre, The University of Sheffield. They were maintained in 11-l, free-draining pots containing M3 compost (Levington) and perlite (Sinclair), mixed in a 2:1 volume ratio, under well-watered and suitably fertilized (Scotts Evergreen Lawn Food; The Scotts Company) conditions. They grew in ambient CO_2_ and received 15 h daylight at the time of harvesting, with light intensities at the leaf levels measured using a light meter (LI-250A; LI-COR) at ≥500 and ≤12 μmol m^−2^ s^−1^ photosynthetic photon flux density for light and dark photoperiods, respectively. After a minimum of 30 days under the above conditions, 1.28 cm^2^ mid-sections of leaf tissues were harvested after 7.5 h of exposure to daylight and after 7.5 h of dark, flash-frozen in liquid nitrogen, and disrupted by grinding to homogeneity when frozen using a mortar and pestle. To extract their protein contents, the ground tissues were resuspended in Extraction Buffer (200 mM bicine-KOH, pH 9.8, 5 mM dithiothreitol [DTT], with 1 tablet cOmplete protease inhibitor cocktail tablets [Roche] per 10 ml), snap-frozen in aliquots, stored in −80 °C, and used within 30 days. Proteins were colorimetrically quantitated (λ = 562 nm) via the BSA assay (Pierce) with BSA standards.

Enzyme assays were conducted as described above for the cloned genes, but only *K*_M_(PEP) values were collected from the in vivo samples as absolute PEPC and ${\text{HCO}}_{3}^{-}$ concentrations are difficult to estimate from leaf extracts. The in vivo measurements of non-C_4_ accessions are difficult to compare with cloned genes, as non-C_4_ individuals express multiple isoforms at low levels (Dunning et al. 2017). Focusing on the C_4_ accessions, there is an overall good correlation between the in vivo and in vitro measurements of *K*_M_(PEP), although more variation exists in leaf extracts (supplementary fig. S2 and table S2, Supplementary Material online). These results indicate that, despite important posttranscriptional regulations of PEPC (Jiao and Chollet 1991; Chollet et al. 1996; O’Leary et al. 2011), our comparisons of kinetic parameters are physiologically meaningful. 

## Supplementary Material

Supplementary data are available at *Molecular Biology and Evolution* online.

## Supplementary Material

msaa143_supplementary_data
