# Supplementary material for: Lateral Gene Transfer Acts As an Evolutionary Shortcut to Efficient C4 Biochemistry
Source: Mol Biol Evol. 2020 Jun 10;37(11):3094–104. doi: 10.1093/molbev/msaa143 (PMC7751175; doi:10.1093/molbev/msaa143)
Supplement: msaa143_supplementary_data [file msaa143_supplementary_data.zip › Phansopa_supp.pdf]

## Supplementary Material for:

## “Lateral gene transfer acts as an evolutionary shortcut to efficient $C_4$ biochemistry”

by Chatchawal Phansopa, Luke T. Dunning, James D. Reid, and Pascal-Antoine Christin[1,3]

This Supplementary Material contains two figures, two tables, and one dataset:

**Figure S1. Phylogenetic trees for distinct groups of PEPC genes.**

**Figure S2. Comparison of $K_M$(PEP) between recombinant enzymes and leaf extracts.**

**Table S1. Properties measured for the different enzymes.**

**Table S2. $K_M$(PEP) values obtained for recombinant proteins and leaf extracts from the same plants.**

**Dataset 1. Sequences of analysed proteins, in fasta format (provided as separate text file).**

**Table S1. Properties measured for the different enzymes.**

| index[a] | category | $k_{cat}$ ($s^{-1}$) | $K_M(HCO_3^-)$ (µM) | $K_M$(PEP) (µM) | $K_I$(MAL) (mM) | $K_I$(ASP) (mM) |
|---|---|---|---|---|---|---|
| 1 | non-$C_4$ | 25.0 ±0.6 | 64.6 ±4.2 | 319.4 ±33.5 | 9.9 ±3.2 | 2.8 ±1.0 |
| 2 | native co-opted 1 | 46.78 ±0.8 | 186.2 ±14.0 | 196.0 ±12.9 | 2.0 ±0.2 | 2.5 ±0.4 |
| 3 | non-$C_4$ | 17.0 ±0.4 | 186.4 ±18.6 | 192.4 ±19.5 | - | - |
| 4 | non-$C_4$ | 21.1 ±0.4 | 124.3 ±6.8 | 181.2 ±14.8 | 0.5 ±0.1 | 0.4 ±0.1 |
| 5 | native co-opted 2 | 47.6 ±0.8 | 185.3 ±12.9 | 188.0 ±12.5 | 3.0 ±0.6 | 1.6 ±0.1 |
| 6 | native co-opted 2 | 69.7 ±1.4 | 240.3 ±32.2 | 324.4 ±23.3 | 4.9 ±0.4 | 1.6 ±0.1 |
| 7 | Donor_M | 78.5 ±3.0 | 567.3 ±43.0 | 920.6 ±99.1 | - | - |
| 8 | LGT:M | 74.5 ±1.7 | 423.0 ±53.8 | 710.2 ±47.2 | 6.0 ±1.6 | 1.1 ±0.1 |
| 9 | LGT:M | 76.9 ±1.9 | 377.6 ±42.2 | 702.4 ±51.7 | 1.4 ±0.2 | 1.5 ±0.3 |
| 10 | donor_A | 88.57 ±3.2 | 882.3 ±83.6 | 686.9 ±72.8 | - | - |
| 11 | LGT:A | 80.0 ±2.0 | 662.3 ±61.5 | 698.6 ±50.4 | 2.2 ±0.2 | 2.6 ±0.3 |
| 12 | donor_C | 78.3 ±2.4 | 1130.7 ±87.6 | 611.7 ±55.9 | - | - |
| 13 | LGT:C | 80.5 ±1.9 | 1032.4 ±75.9 | 681.2 ±47.7 | 5.8 ±2.3 | 3.1 ±0.5 |
| 14 | LGT:C | 83.1 ±2.2 | 1065.7 ±86.6 | 692.8 ±53.0 | 5.5 ±1.6 | 3.0 ±0.4 |

[a] isoforms are numbered as in Table 1.

**Table S2. $K_M$(PEP) values obtained for recombinant proteins and leaf extracts from the same plants.**

| index[a] | category | leaf day[b] | leaf night[b] | recombinant[b] | expression[c] | fraction[d] |
|---|---|---|---|---|---|---|
| 2 | native co-opted 1 | 41.2 ±3.1 | 171.4 ±12.8 | 196.0 ±12.9 | 11153 | 0.85 |
| 5 | native co-opted 2 | 41.8 ±2.5 | 349.5 ±19.4 | 188.0 ±12.5 | 4176 | 0.93 |
| 6 | native co-opted 2 | 97.0 ±10.7 | 397.9 ±41.9 | 324.4 ±23.3 | 5792 | 0.97 |
| 8 | LGT:M | 408.6 ±28.9 | 1289.0 ±167 | 710.2 ±47.2 | 2559 | 0.97 |
| 9 | LGT:M | 592.4 ±62.6 | 1350.0 ±143.7 | 702.4 ±51.7 | 2224 | 0.99 |
| 10 | donor_A | 174.4 ±29.6 | 499.7 ±44.7 | 686.9 ±72.8 | NA | NA |
| 11 | LGT:A | 305.9 ±27.7 | 940.3 ±79.4 | 698.6 ±50.4 | 2613 | 0.96 |
| 13 | LGT:C | 619.8 ±43.7 | 1384 ±156.2 | 681.2 ±47.7 | 3980 | 0.87 |
| 14 | LGT:C | 613.5 ±68.8 | 1424 ±180.9 | 692.8 ±53 | 3218 | 0.79 |

[a] isoforms are numbered as in Table 1; [b] all values are given in μM; [c] average expression of the gene in leaves of individuals from the same population sampled during the day, in reads per million of mappable reads per kilobases (rpkm; Dunning et al. 2017); [d] fraction of the total transcript abundance represented by the cloned gene lineage.

**Figure S1. Phylogenetic trees for distinct groups of PEPC genes.**

The phylogenetic tree of each lineage with some members used for $C_4$ photosynthesis in *Alloteropsis* is shown first with topology and branch lengths inferred from 3$^{rd}$ positions of codons and then with branch lengths estimated from amino acids while keeping the topology fixed. Bootstrap support values are indicated near nodes when greater than 50. Names of individuals using $C_4$ photosynthesis are in red while those of individuals using a weak $C_4$ pathway (see Dunning et al. 2017) are in orange. Putative pseudogenes containing stop codons are indicated with crosses, in black when all alleles are pseudogenes and in grey when a mixture of pseudogenes and functional alleles might co-exist. Names of accessions are as in Dunning et al. 2019a. Genes characterised biochemically are highlighted with symbols matching those in Figs 1, 2 and 3, and numbered as in Table 1.

# *ppc-1P6* (3rd positions)

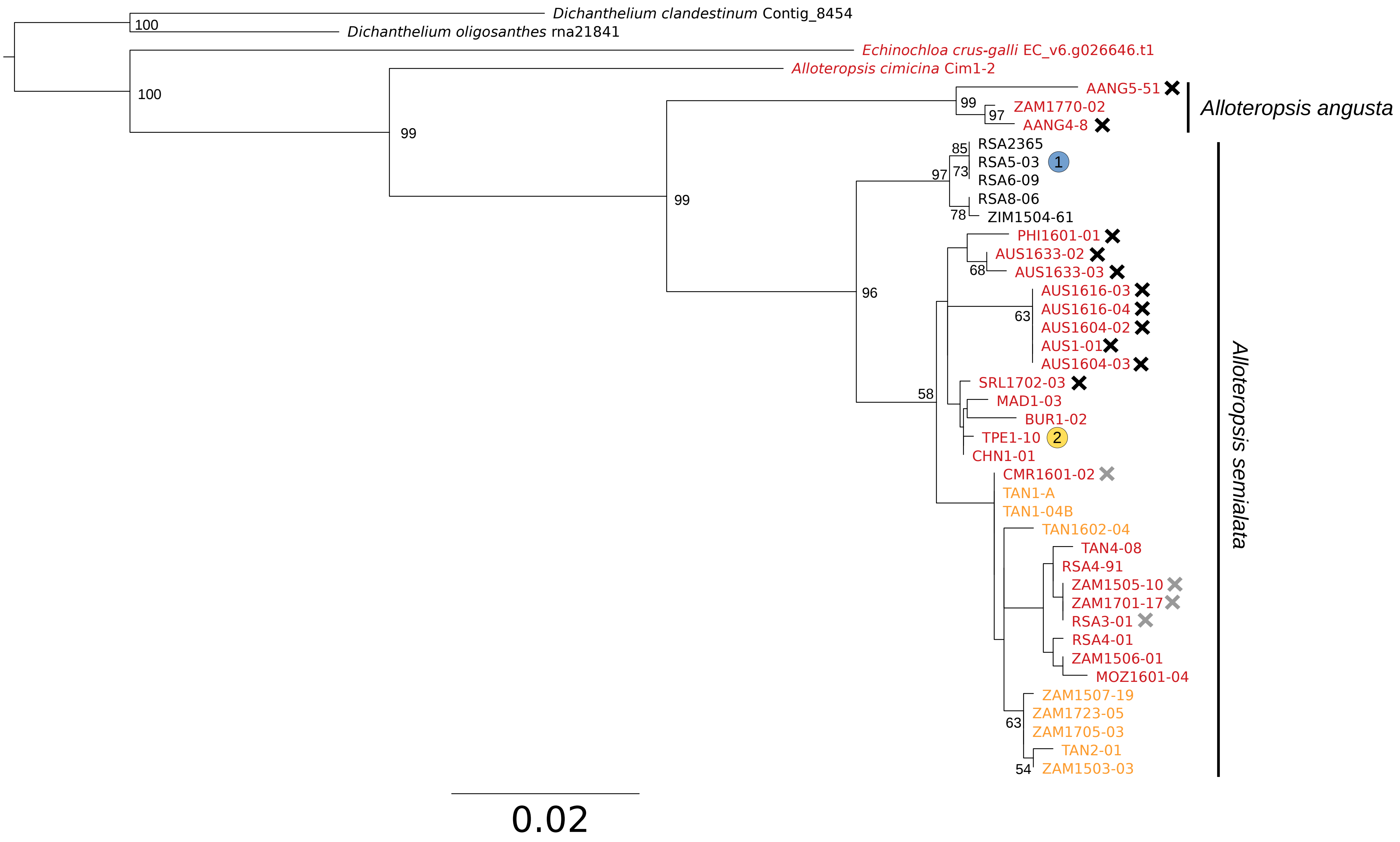

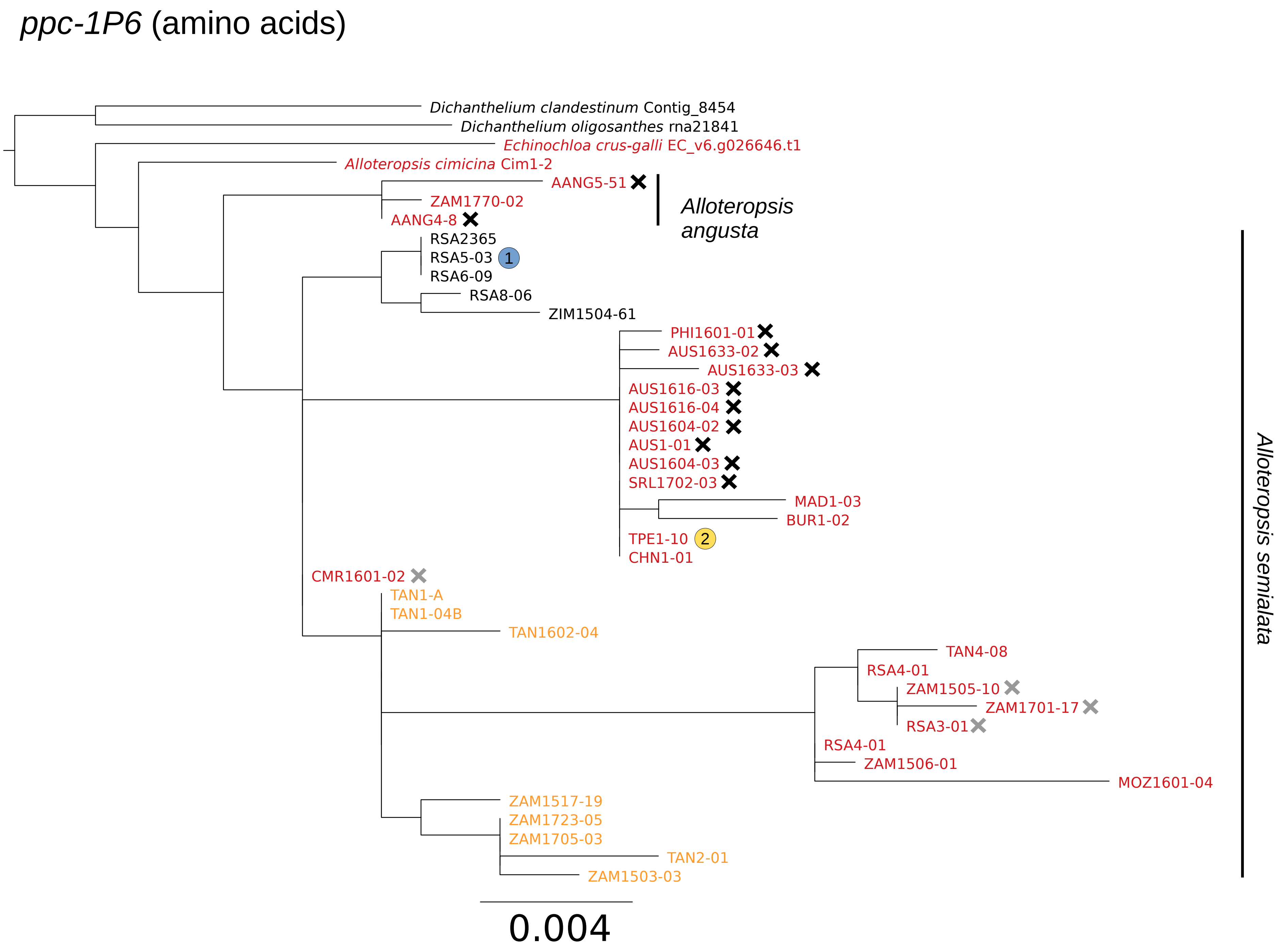
ppc-1P6 (amino acids)
Dichanthelium clandestinum Contig_8454
Dichanthelium oligosanthes rna21841
Echinochloa crus-galli EC_v6.g026646.t1
Alloteropsis cimicina Cim1-2
AANG5-51
ZAM1770-02
AANG4-8
Alloteropsis angusta
RSA2365
RSA5-03
1
RSA6-09
RSA8-06
ZIM1504-61
PHI1601-01
AUS1633-02
AUS1633-03
AUS1616-03
AUS1616-04
AUS1604-02
AUS1-01
AUS1604-03
SRL1702-03
MAD1-03
BUR1-02
TPE1-10
2
CHN1-01
CMR1601-02
TAN1-A
TAN1-04B
TAN1602-04
TAN4-08
RSA4-01
ZAM1505-10
ZAM1701-17
RSA3-01
RSA4-01
ZAM1506-01
MOZ1601-04
ZAM1517-19
ZAM1723-05
ZAM1705-03
TAN2-01
ZAM1503-03
0.004
Alloteropsis semialata

# *ppc-1P3* (3rd positions)

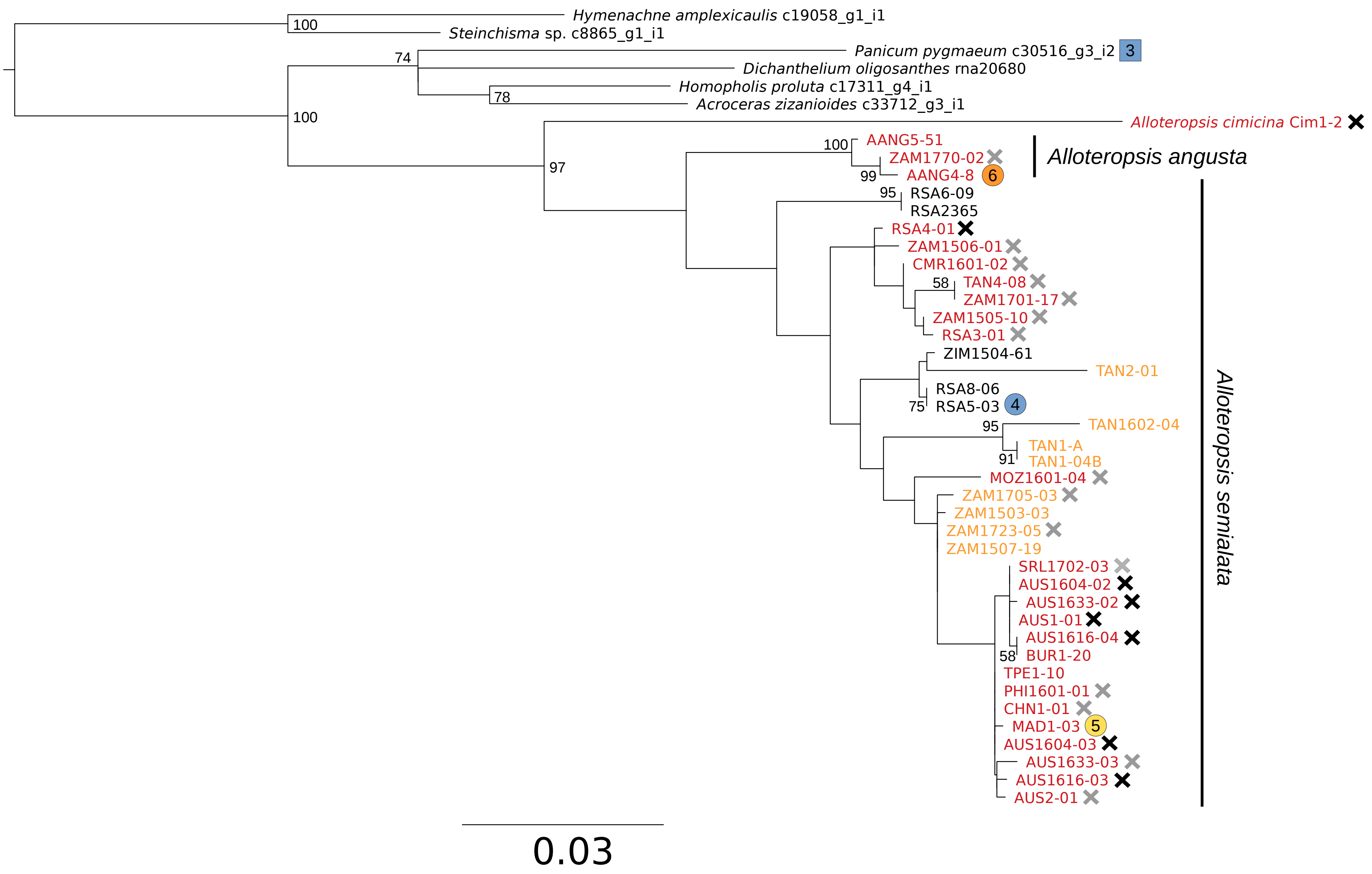

# *ppc-1P3* (amino acids)

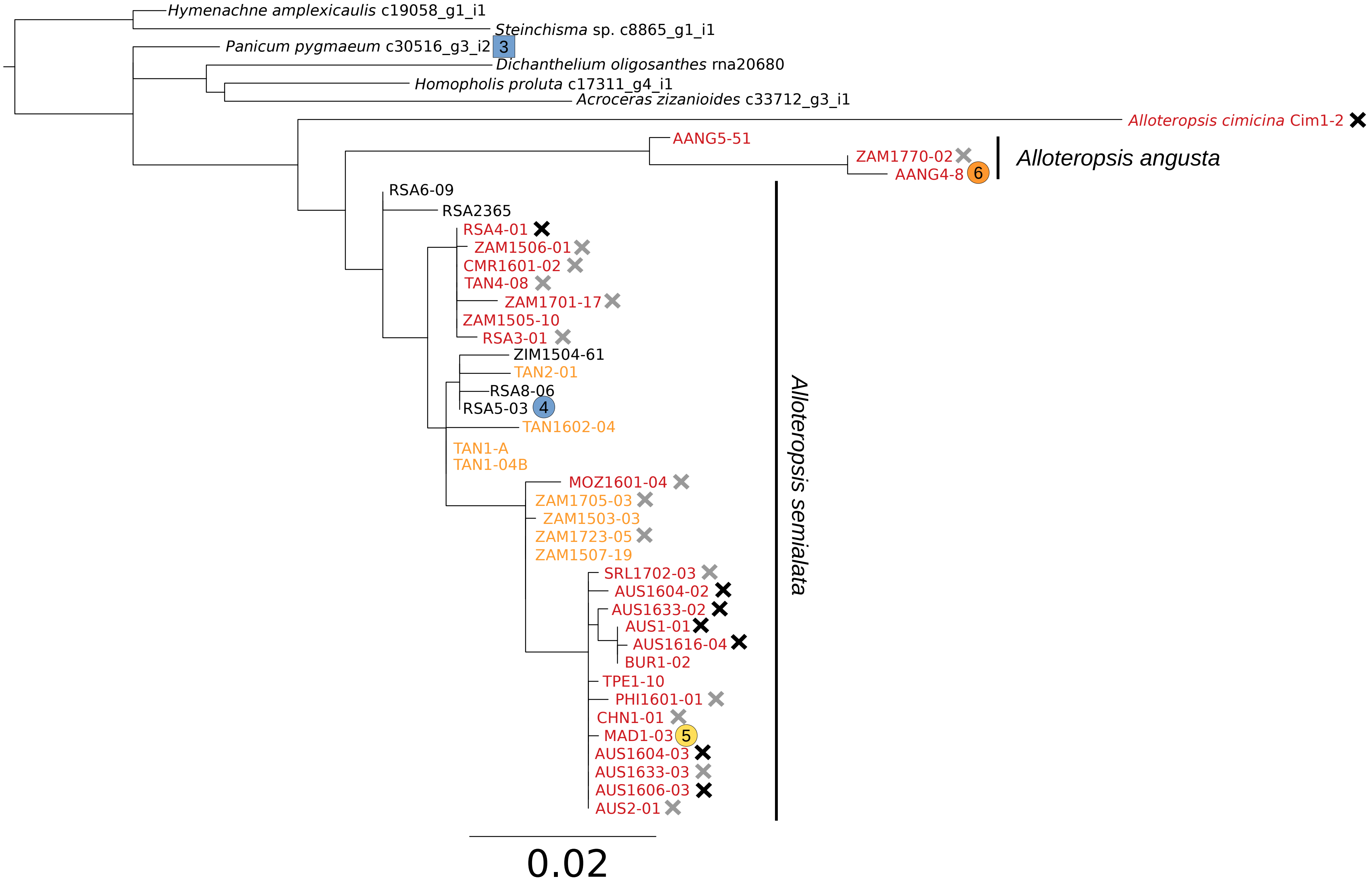

# *ppc-1P3_LGT:M* (3rd positions)

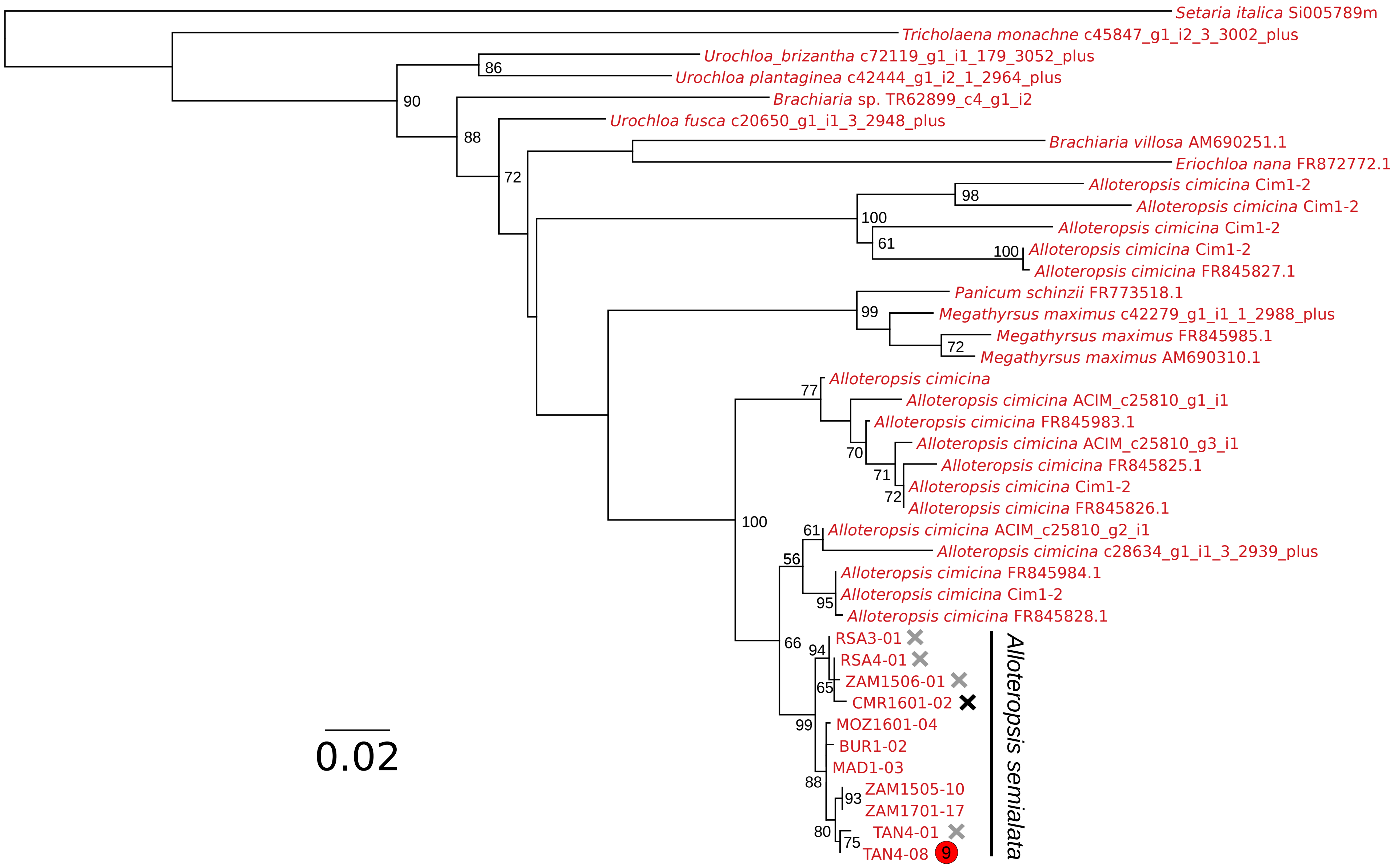

# *ppc-1P3_LGT:M* (amino acids)

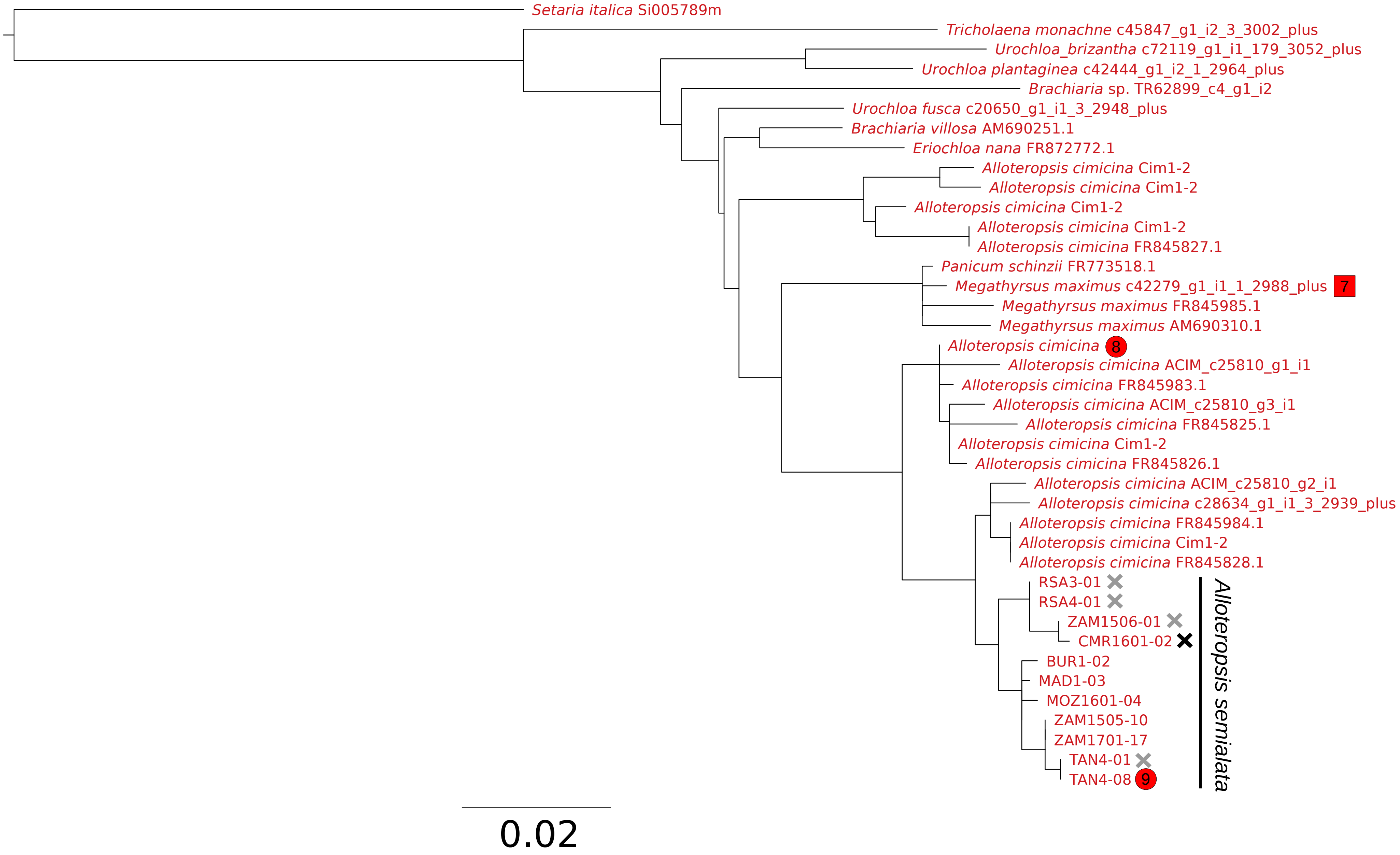

*ppc-1P3_LGT:A* (3rd positions)

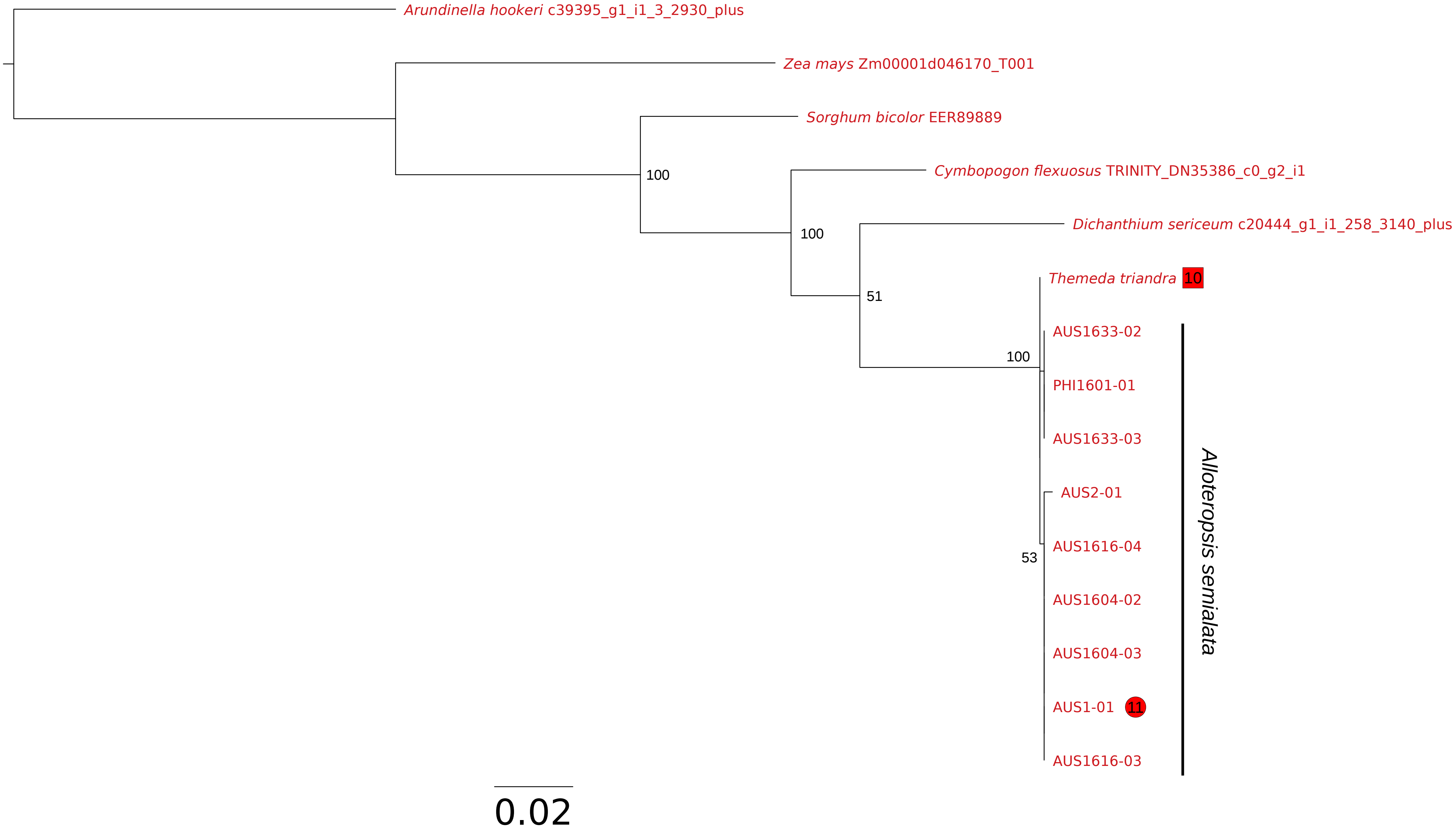

# *ppc-1P3_LGT:A* (amino acids)

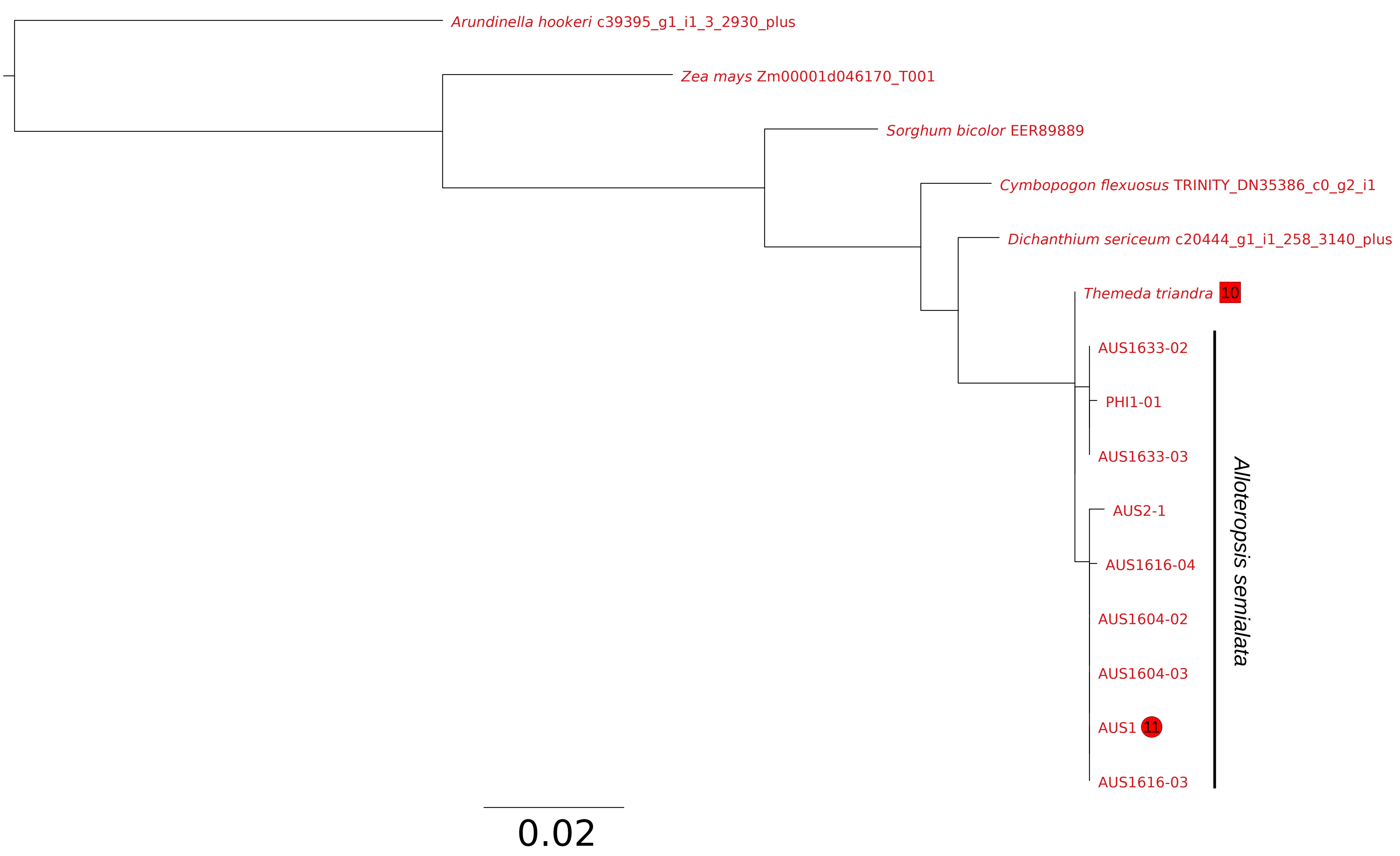

# *ppc-1P3_LGT:C* (3rd positions)

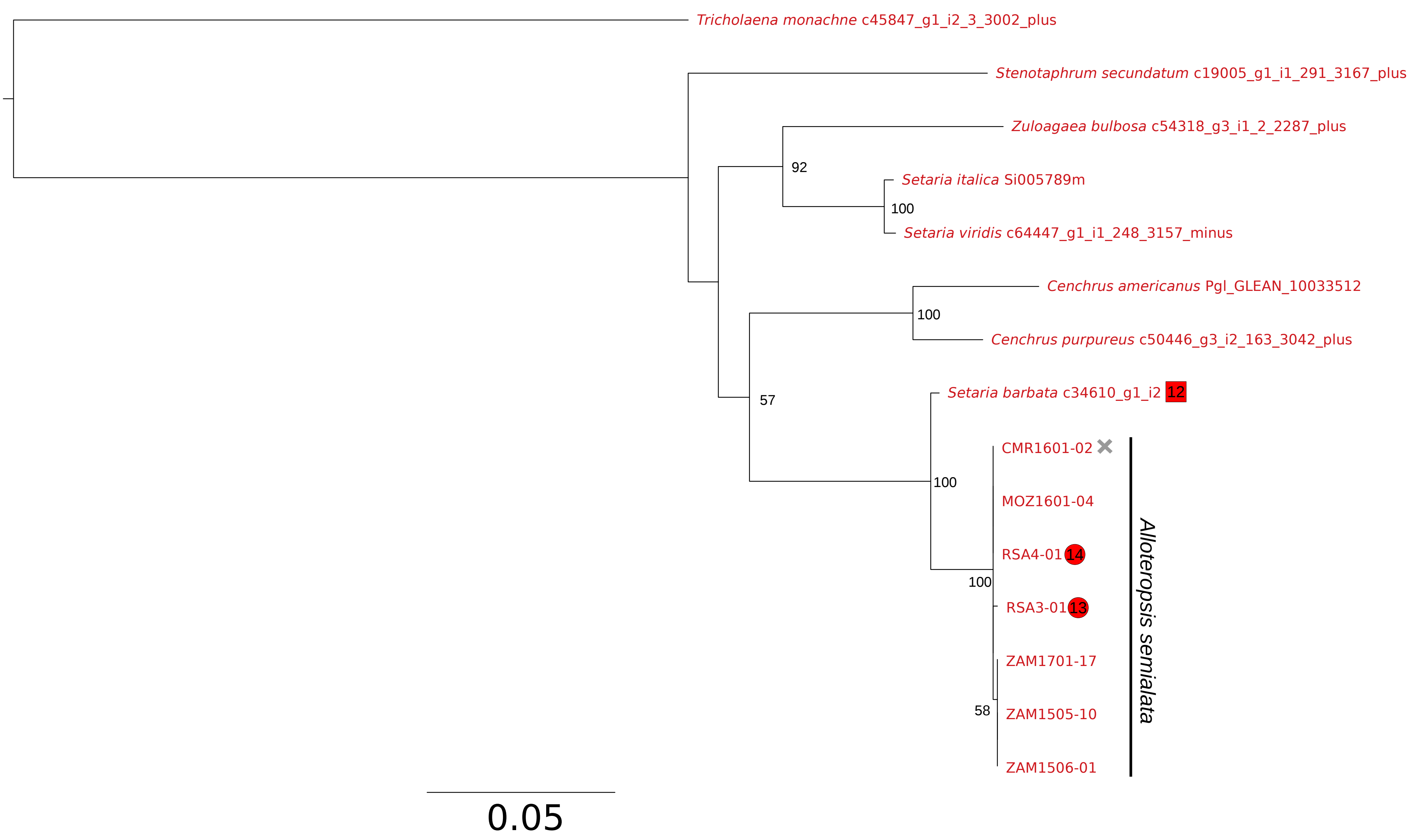

# *ppc-1P3_LGT:C* (amino acids)

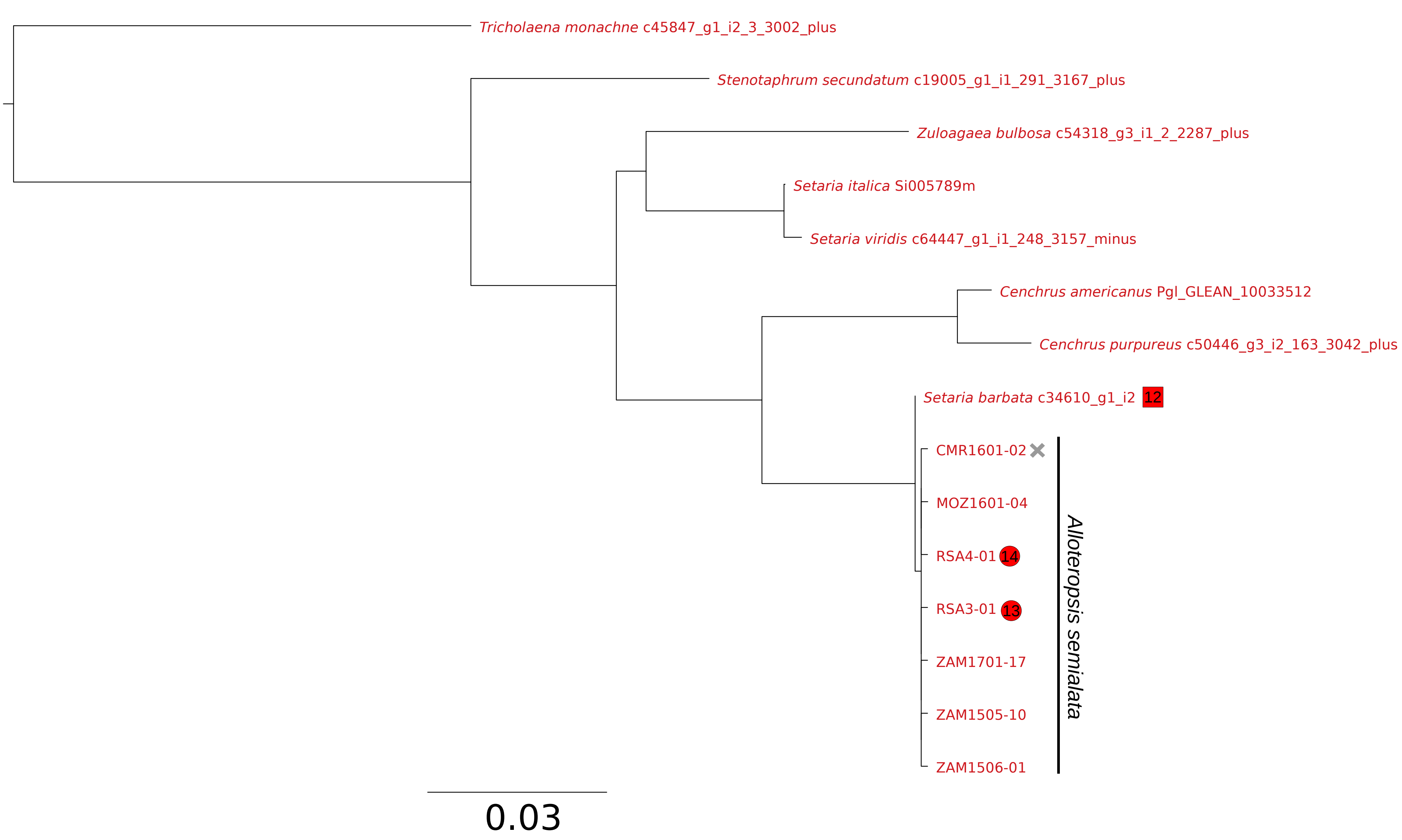

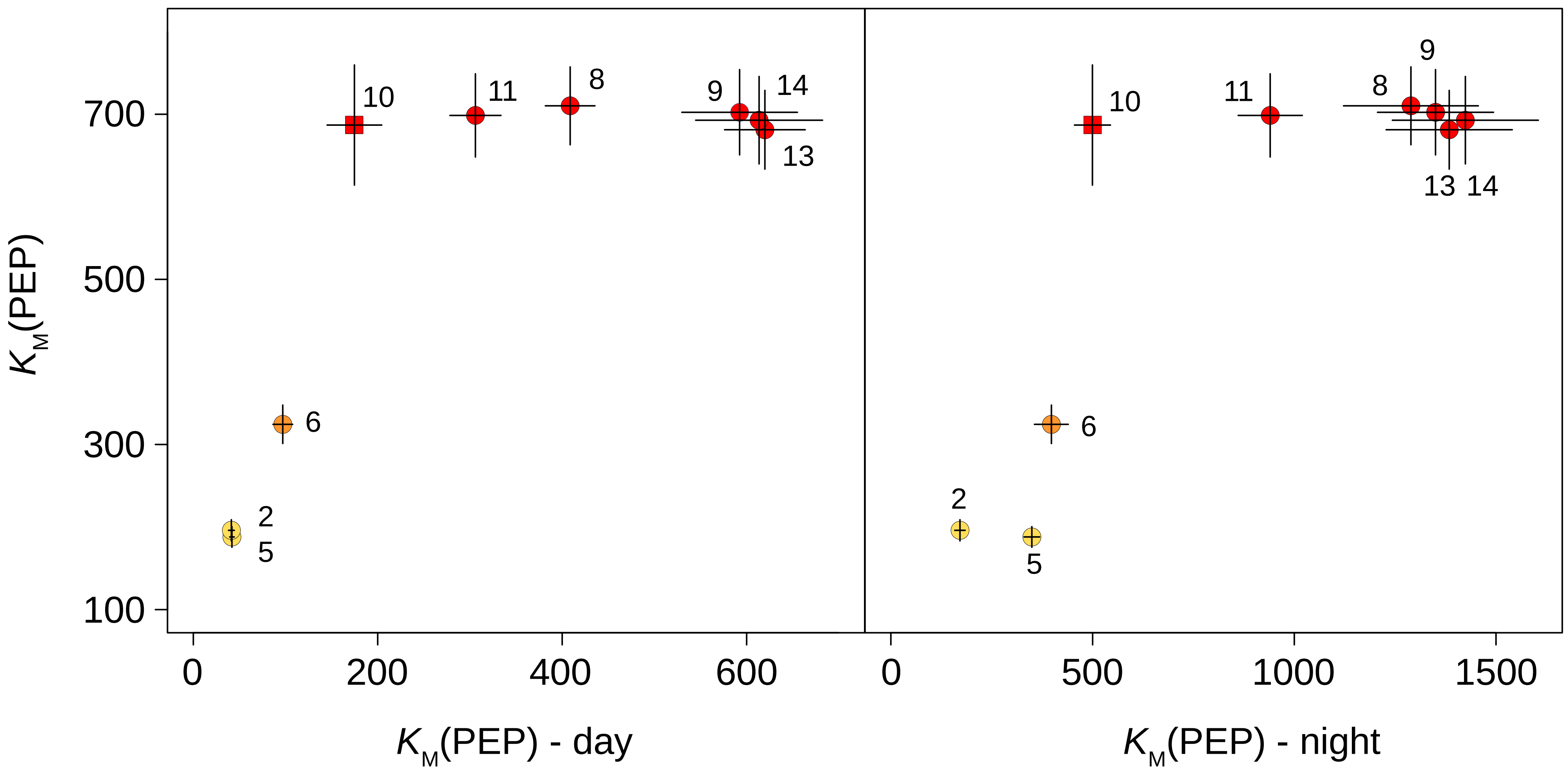


**Figure S2. Comparison of Km(PEP) between recombinant enzymes and leaf extracts.**

The values for each recombinant enzyme are compared to those of the plants expressing it at high values, with leaves samples either during the day or during the night. Samples are numbered as in Table 1, with symbols matching those in Figs 3 and 4. For details, see Table S3.
